# Calcineurin Mediates Synaptic Scaling Via Synaptic Trafficking of Ca^2+^-Permeable AMPA Receptors

**DOI:** 10.1371/journal.pbio.1001900

**Published:** 2014-07-01

**Authors:** Seonil Kim, Edward B. Ziff

**Affiliations:** Department of Biochemistry and Molecular Pharmacology, New York University Langone Medical Center, New York, New York, United States of America; California Institute of Technology, United States of America

## Abstract

Homeostatic synaptic plasticity is a negative-feedback mechanism for compensating excessive excitation or inhibition of neuronal activity. When neuronal activity is chronically suppressed, neurons increase synaptic strength across all affected synapses via synaptic scaling. One mechanism for this change is alteration of synaptic AMPA receptor (AMPAR) accumulation. Although decreased intracellular Ca^2+^ levels caused by chronic inhibition of neuronal activity are believed to be an important trigger of synaptic scaling, the mechanism of Ca^2+^-mediated AMPAR-dependent synaptic scaling is not yet understood. Here, we use dissociated mouse cortical neurons and employ Ca^2+^ imaging, electrophysiological, cell biological, and biochemical approaches to describe a novel mechanism in which homeostasis of Ca^2+^ signaling modulates activity deprivation-induced synaptic scaling by three steps: (1) suppression of neuronal activity decreases somatic Ca^2+^ signals; (2) reduced activity of calcineurin, a Ca^2+^-dependent serine/threonine phosphatase, increases synaptic expression of Ca^2+^-permeable AMPARs (CPARs) by stabilizing GluA1 phosphorylation; and (3) Ca^2+^ influx via CPARs restores CREB phosphorylation as a homeostatic response by Ca^2+^-induced Ca^2+^ release from the ER. Therefore, we suggest that synaptic scaling not only maintains neuronal stability by increasing postsynaptic strength but also maintains nuclear Ca^2+^ signaling by synaptic expression of CPARs and ER Ca^2+^ propagation.

## Abstract 2

Kim and Ziff examine the molecular mechanism of synaptic scaling, showing that inhibition of neuronal excitability reduces calcium influx into neurons, resulting in decreased calcineurin activity. This leads to increased surface expression of calcium-permeable AMPA receptors as a homeostatic response.

## Author Summary

Synaptic scaling is a form of homeostatic plasticity that normalizes the strength of synapses (the structure that allows nerve cells to communicate) and is triggered by chronic inhibition of neuronal activity. Although extensive studies have been conducted, the molecular mechanism of this synaptic adaptation is not understood. Using cultured cortical neurons, we show that chronic inhibition of neuronal activity reduces calcium influx into neurons, which, in turn, decreases the activity of the calcium-dependent phosphatase calcineurin. These changes lead to an increase in GluA1-containing, calcium-permeable AMPA receptors, which mediate communication at the synapse. Newly inserted calcium-permeable AMPA receptors restore calcium currents, which enhance synaptic strength and recover calcium signaling. We also show that inhibition or activation of calcineurin activity is sufficient to induce or block synaptic scaling, respectively, suggesting that calcineurin is an important mediator of homeostatic synaptic plasticity. Taken together, our findings show that synaptic scaling is a homeostatic process that not only enhances synaptic transmission but also maintains calcium signaling in neurons under activity deprivation.

## Introduction

Synaptic scaling, a form of homeostatic synaptic plasticity, is a negative feedback process that stabilizes neuronal activity in response to changes in synaptic strength by altering various aspects of neuronal function [1]. It has been implicated in neurodevelopment and in neurological disorders [2]–[5]. One of the mechanisms underlying synaptic scaling is the regulation of synaptic strength through control of delivery or retention of AMPARs at synapses [1]. During homeostatic adaptation, synaptic AMPARs are increased or reduced in response to activity deprivation or overexcitation, respectively, by altering AMPAR synaptic insertion and internalization [6]. Synaptic adaptation can be global and multiplicative, which is important for preserving the relative strength differences between synapses. Because each synapse strength is multiplied or divided by the same factor, each synaptic strength is increased or decreased in proportion to its initial strength [7]. Synaptic scaling is also induced by synapse-specific processes, providing local control of synaptic strength [8]. Numerous treatments that induce homeostatic regulation but differ in their experimental conditions have been reported. Nonetheless, the homeostatic plasticity mechanism is still not well understood. Here, we describe a novel mechanism in which activity deprivation induces synaptic scaling by a calcineurin-mediated process.

AMPARs are the major excitatory postsynaptic glutamate receptor in the central nervous system and consist of four subunits (GluA1–4) [9]. There are two general types of AMPARs formed through combination of these subunits, Ca^2+^-impermeable GluA2-containing and Ca^2+^-permeable, GluA2-lacking/GluA1-containing receptors [10]. Ca^2+^-permeable AMPARs (CPARs) are generally sensitive to polyamine block, although there is a third class of AMPARs that are Ca^2+^-permeable but insensitive to polyamines [11]. The GluA1 and GluA2 AMPAR subunits can assemble channels with markedly different electrophysiological and trafficking properties [10],[11] and both GluA1 and GluA2 can contribute to homeostatic synaptic plasticity [1],[12],[13]. Phosphorylation of GluA1 within its intracellular carboxyl-terminal domain can regulate AMPAR membrane trafficking and channel open probability [14]. Phosphorylation of serine 845 in GluA1 [pGluA1(S845)] is important for activity-dependent trafficking of GluA1-containing AMPARs, and cAMP-dependent protein kinase A (PKA) and cGMP-dependent protein kinase II (cGKII) can mediate this phosphorylation [14],[15]. The Ca^2+^/calmodulin-dependent protein phosphatase, calcineurin, dephosphorylates pGluA1(S845), which enables GluA1-containing AMPARs to be endocytosed from the plasma membrane during long-term depression [16],[17]. Therefore, activity-dependent GluA1 phosphorylation can play critical roles in GluA1 synaptic trafficking and forming CPARs in synapses.

The most studied experimental system for synaptic scaling is the inhibition of neuronal activity by TTX (tetrodotoxin), which blocks sodium channels and thereby inhibits action potentials. TTX-dependent chronic inhibition of action potentials results in an increase in the strength of synaptic transmission as a compensatory process that can be measured by increases in AMPAR-mediated miniature excitatory postsynaptic currents (mEPSCs) [18]. Recent studies suggest that TTX reduces somatic Ca^2+^ influx and inhibits activation of Ca^2+^/calmodulin-dependent protein kinase IV (CaMKIV), which promotes synaptic scaling [19]. CaMKs are important for Ca^2+^-dependent synaptic plasticity [20], and inhibition of Ca^2+^ influx is sufficient to induce AMPAR-mediated synaptic scaling [21],[22]. This suggests that reduction of CaMK activation and downstream signaling by activity deprivation-induced inhibition of Ca^2+^ influx can play a critical role in AMPAR-dependent homeostatic scaling, yet there is no complete molecular mechanism linking activity-dependent Ca^2+^ signals and homeostatic regulation of AMPARs.

Here, we focus on the role of synaptic Ca^2+^ and calcineurin in synaptic scaling. We show that activity suppression reduces Ca^2+^ influx in neurons, which in turn decreases the activity of calcineurin. This stabilizes pGluA1(S845), which increases synaptic CPARs. This increases synaptic strength as a compensatory response to activity deprivation and restores synapse-to-nucleus Ca^2+^ signaling via ER Ca^2+^ wave propagation. Thus, we conclude that synaptic scaling via calcineurin and CPARs provides a means to maintain not only synaptic activity but also Ca^2+^ signaling as a homeostatic response.

## Results

### TTX-Induced Multiplicative Synaptic Scaling Depends on CPARs

**Figure 1:**
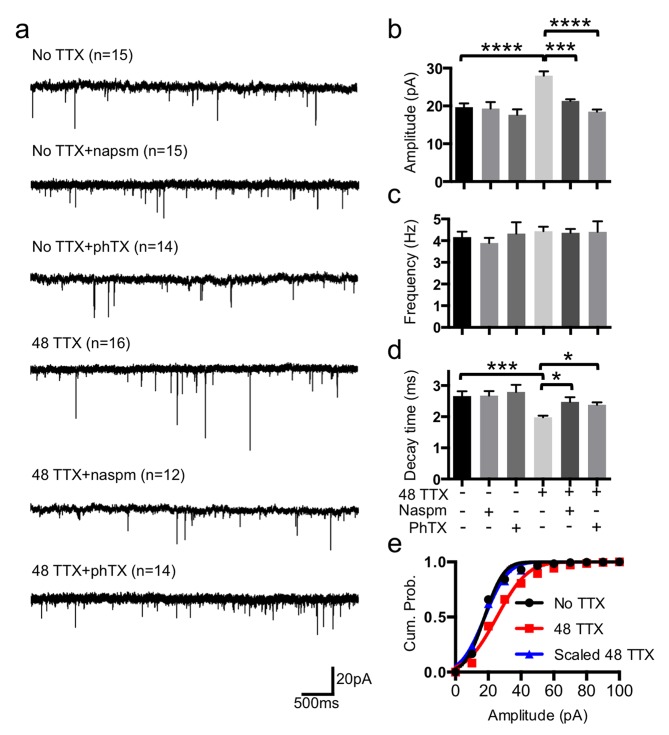
TTX-induced multiplicative synaptic scaling mediated by CPARs. (a) Representative traces of mEPSC recordings in each condition (*n* = number of cells). (b) Average mEPSC amplitude (***p<.001 and ****p<.0001, one-way ANOVA, uncorrected Fisher's LSD). (c) Average mEPSC frequency. (d) Average decay time (peak to 10%) (*p<.05 and ***p<.001, one-way ANOVA, uncorrected Fisher's LSD). (e) Average cumulative probability of mEPSC amplitude. TTX distribution is significantly different from no TTX (*p* = .015, K-S test). Distribution of TTX scaled down by a factor of 1.42 fitted to no TTX distribution (*p* = .935, K-S test).

To confirm activity-dependent homeostatic scaling, we studied spontaneous synaptic transmission by measuring mEPSCs in DIV14–17 cultured mouse cortical neurons (Figure 1a) and found that treatment for 48 h with 2 µM TTX significantly increased average mEPSC amplitude (no TTX, 19.68±0.99 pA and 48 TTX, 28.01±1.12 pA, *p*<.0001) (Figure 1b) consistent with the previous finding [23], whereas mEPSC frequency was not altered (Figure 1c). Importantly, cumulative probability distributions of the mEPSC amplitude were uniformly increased by TTX treatment, and the increase in the amplitude with TTX treatment was multiplicative (Figure 1e). There was a significant decrease in mEPSC decay time (peak to 10%) with TTX treatment (no TTX, 2.66±0.15 ms and 48 TTX, 1.98±0.05 ms, *p* = .0008) (Figure 1d). Because CPARs show a shorter decay time [21],[24], we used 20 µM naspm (1-naphthyl acetyl spermine) or 5 µM PhTX (philanthotoxin-74), blockers of CPARs, to determine if CPARs were responsible for the TTX-mediated increase of the amplitude (Figure 1a). Consistent with previous findings [21],[23],[25], naspm and PhTX treatment significantly reduced the TTX-induced increase in amplitude (48 TTX, 28.01±1.12 pA; 48 TTX+naspm, 21.31±0.44 pA, *p* = .0002; and 48 TTX+PhTX, 18.50±0.58 pA, *p*<.0001) (Figure 1b), but frequency was not affected (Figure 1c). Naspm and PhTX also significantly increased decay time (48 TTX, 1.98±0.05 ms; 48 TTX+naspm, 2.47±0.15 ms, *p* = .0186; and 48 TTX+PhTX, 2.38±0.07 ms, *p* = .0453) (Figure 1d) as found previously [21]. CPAR inhibitors had no effects on mEPSCs of neurons in the absence of TTX treatment, suggesting that CPARs made no contribution under the basal condition (Figure 1). Thus, TTX treatment induced CPAR-mediated multiplicative synaptic scaling.

### pGluA1(S845) Is Required for TTX-Induced Synaptic Scaling

**Figure 2:**
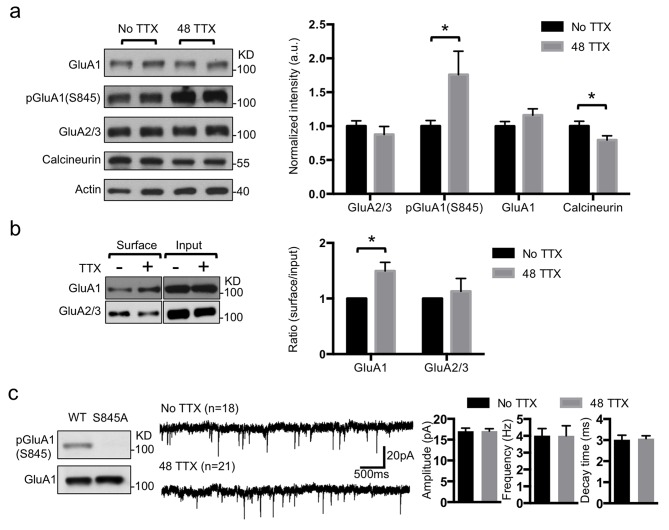
TTX increased GluA1 surface trafficking by phosphorylation but decreased synaptic calcineurin levels. (a) Representative immunoblots and quantitative analysis of synaptosomes from cultured cortical neurons in the presence and absence of TTX treatment (*n* = 6 experiments, *p<.05, unpaired two-tailed Student's *t* test). (b) Representative immunoblots of surface biotinylation and a summary graph in the presence and absence of TTX treatment (*n* = 3 experiments, *p<.05, unpaired two-tailed Student's *t* test). (c) Representative immunoblots showing deficiency of phosphorylation in GluA1 S845A mutant neurons (*n* = 3 experiments). Representative traces of mEPSCs in the presence or absence of TTX (*n* = number of cells). Average mEPSC amplitude, frequency, and decay time (peak to 10%).

Because pGluA1(S845) is required not only for homeostatic scaling in the visual cortex [26] but also for maintaining CPARs on the synaptic membrane [27], we measured the effects of TTX on pGluA1(S845) levels by purifying synaptosomes from TTX-treated neurons and measuring protein and phosphorylation levels of AMPAR subunits. TTX treatment significantly increased pGluA1(S845) (*p* = .024), whereas total GluA1 and GluA2/3 levels were not changed (Figure 2a). We further determined that surface GluA1 levels were increased (*p* = .0127) after TTX treatment, whereas surface GluA2/3 was not altered (Figure 2b). We next analyzed mutant GluA1 (GluA1 S845A, unable to be phosphorylated on serine 845) using GluA1 S845A knock-in mice [28] and found that TTX treatment was unable to induce synaptic scaling in neurons from the mutant mouse (Figure 2c). This suggested that TTX treatment enhanced GluA1 surface trafficking by increasing pGluA1(S845). This newly trafficked GluA1 could be in the form of CPARs that promote synaptic scaling.

### TTX Treatment Reduces Calcineurin Activity in a Time-Dependent Manner

**Figure 3:**
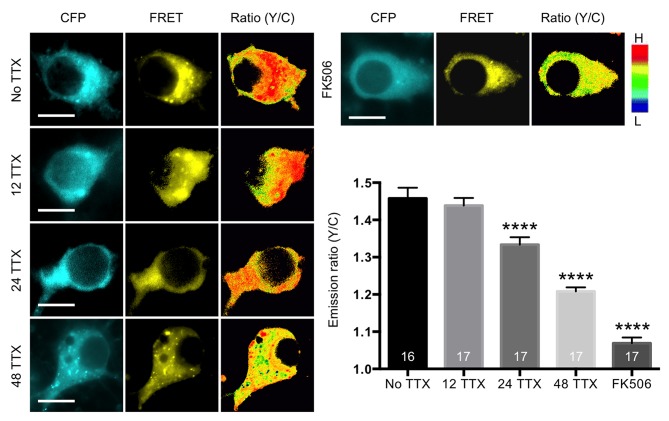
TTX reduced *in vivo* calcineurin activity in a time-dependent manner. Representative images of CFP channel, FRET channel, and pseudocolored emission ratio (Y/C) in each condition [blue (L), low emission ratio; red (H), high emission ratio]. Scale bar is 10 µm. A summary graph showing average of emission ratio (Y/C) in each condition (*n* = number of cells) (****p<.0001, one-way ANOVA, uncorrected Fisher's LSD).

Increasing pGluA1(S845) can be achieved either by enhancing kinase activity or by decreasing phosphatase activity. A-kinase anchoring protein (AKAP) and SAP97 form a protein complex with GluA1 that tethers PKA and calcineurin, which regulate channel functions, respectively, through GluA1 phosphorylation and dephosphorylation [29],[30]. Therefore, reduction of calcineurin activity is a candidate for mediating an increase of pGluA1(S845) in response to the TTX-induced reduction of Ca^2+^ influx. We found that calcineurin protein levels were significantly decreased (*p* = .0414) in synaptosomes following TTX treatment (Figure 2a). To measure *in vivo* calcineurin activity directly, we used a fluorescence resonance energy transfer (FRET)-based calcineurin activity sensor that utilizes a calcineurin activity-dependent molecular switch based on the N-terminal regulatory domain of nuclear factor of activated T cells (NFAT) as a specific substrate, which was inserted between CFP and YFP [31]. Inhibition of calcineurin activity by 12 h treatment with 5 µM FK506, which forms a drug-immunophilin complex that is a highly specific inhibitor for calcineurin [32], significantly decreased FRET activity (assayed by measuring the emission ratio) as compared with that under the basal condition (no TTX, 1.45±0.02 and FK506, 1.06±0.01, *p*<.0001), which confirmed that the reporter detected calcineurin activity (Figure 3). Calcineurin activity was significantly decreased after 24 h TTX treatment and further reduced after 48 h TTX treatment, whereas 12 h TTX had no effect on the emission ratio (12 TTX, 1.43±0.02; 24 TTX, 1.33±0.02, *p*<.0001; and 48 TTX, 1.20±0.01, *p*<.0001) (Figure 3). This suggested that chronic inhibition of neuronal activity decreased calcineurin activity in a time-dependent manner and lowered synaptic calcineurin levels.

### Loss of Calcineurin Activity Is Sufficient to Induce CPAR-Dependent Synaptic Scaling in the Absence of TTX Treatment

**Figure 4:**
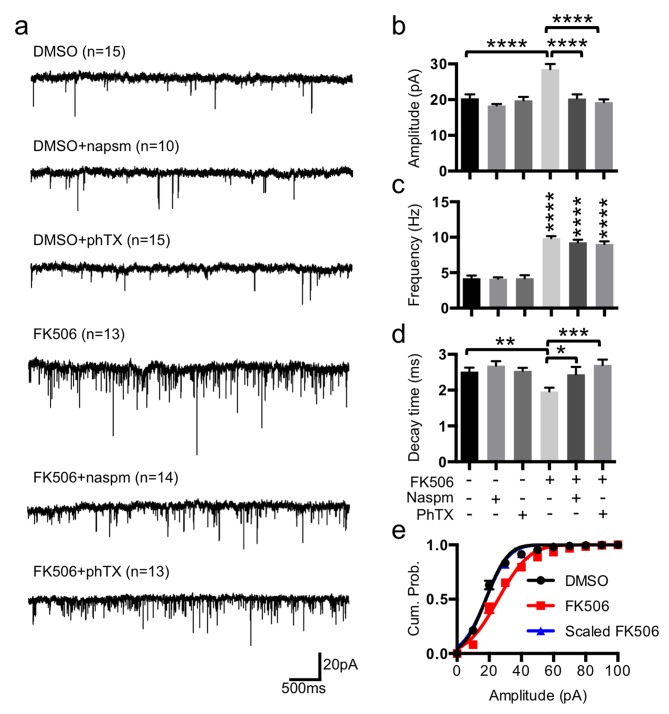
Inhibition of calcineurin activity induced a multiplicative pharmacologic form of synaptic scaling mediated by CPARs. (a) Representative traces of mEPSCs in each condition (*n* = number of cells). (b) Average mEPSC amplitude (****p<.0001, one-way ANOVA, uncorrected Fisher's LSD). (c) Average mEPSC frequency (****p<.0001, one-way ANOVA, uncorrected Fisher's LSD). (d) Average decay time (peak to 10%) (*p<.05, **p<.01, and ***p<.001, one-way ANOVA, uncorrected Fisher's LSD). (e) Average cumulative probability of mEPSC amplitude. FK506 distribution is significantly different from DMSO (*p* = .031, K-S test). Distribution of FK506 scaled down by a factor of 1.44 fitted to DMSO distribution (*p* = .984, K-S test).

**Figure 5:**
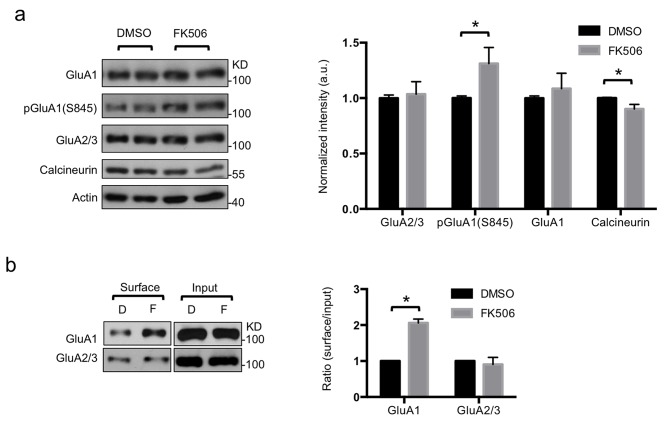
Inhibition of calcineurin activity increased GluA1 surface trafficking by phosphorylation but decreases calcineurin. (a) Representative immunoblots and quantitative analysis of synaptosomes from cultured cortical neurons in the presence and absence of FK506 treatment (*n* = 5 experiments, *p<.05, unpaired two-tailed Student's *t* test). (b) Representative immunoblots of surface biotinylation (D, DMSO; F, FK506) and a summary graph in the presence or absence of FK506 treatment (*n* = 6 experiments, *p<.05, unpaired two-tailed Student's *t* test).

Calcineurin inhibition affects both mEPSC frequency and amplitude [33],[34] and stabilizes pGluA1(S845) [35], and reduction of cytoplasmic Ca^2+^ lowers calcineurin activity, followed by enhancement of GluA1-containing AMPAR-mediated transmission [36]. To determine whether inhibition of calcineurin was sufficient for inducing a pharmacologic form of synaptic scaling in the absence of TTX treatment, we next blocked calcineurin activity by 12 h treatment with 5 µM FK506 and measured mEPSCs (Figure 4a). FK506 treatment significantly increased mEPSC amplitude compared with DMSO treatment (DMSO, 20.38±1.08 pA and FK506, 28.54±1.41 pA, *p*<.0001) (Figure 4b). Consistent with a previous study showing that inhibition of calcineurin increases mEPSC frequency [33],[34] through calcineurin modulation of presynaptic activity [37], we found increased mEPSC frequency in FK506-treated neurons (DMSO, 4.21±0.37 Hz and FK506, 9.88±0.27 Hz, *p*<.0001) (Figure 4c). Furthermore, the mEPSC decay time in FK506-treated neurons was significantly faster (DMSO, 2.51±0.10 ms and FK506, 1.96±0.10 ms, *p* = .0047), suggesting that CPARs mediated the scaling induced by FK506 (Figure 4d). Moreover, cumulative probability distributions were uniformly shifted by FK506, and the increase in the amplitude was multiplicative (Figure 4e). We confirmed CPAR-mediated scaling in FK506-treated neurons by adding naspm or PhTX (Figure 4a), which caused a significant reduction of mEPSC amplitude (FK506, 28.54±1.41 pA; FK506+naspm, 20.31±1.14 pA, *p*<.0001; and FK506+PhTX, 19.33±0.76 pA, *p*<.0001), whereas no effect was observed following naspm or PhTX treatment of DMSO-treated neurons (Figure 4b). There were no significant changes in mEPSC frequency after napsm or PhTX treatment of either DMSO or FK506-treated neurons (Figure 4c). Moreover, the FK506-induced change in decay time was reversed by naspm and PhTX only for the FK506-treated neurons (FK506, 1.96±0.10 ms; FK506+naspm, 2.44±0.20 ms, *p* = .0152; and FK506+PhTX, 2.70±0.15 ms, *p* = .0003) (Figure 4d). Similar to the effects of 48 h TTX treatment, FK506 treatment increased pGluA1(S845) (*p* = .0474) in synaptosomes, whereas total GluA2/3 and GluA1 levels were not altered (Figure 5a). Surface GluA1 was significantly elevated with FK506 treatment (*p*<.0001) (Figure 5b). Moreover, FK506 treatment significantly reduced calcineurin levels in synaptosomes (*p* = .0299) (Figure 5a). These results indicated that inhibition of calcineurin by FK506 was sufficient to induce synaptic trafficking of CPARs by increasing pGluA1(S845) and that FK506 could produce a pharmacologic form of synaptic scaling without TTX-mediated activity deprivation.

### Constitutively Active Calcineurin Mutant Inhibits TTX-Induced Synaptic Scaling

**Figure 6:**
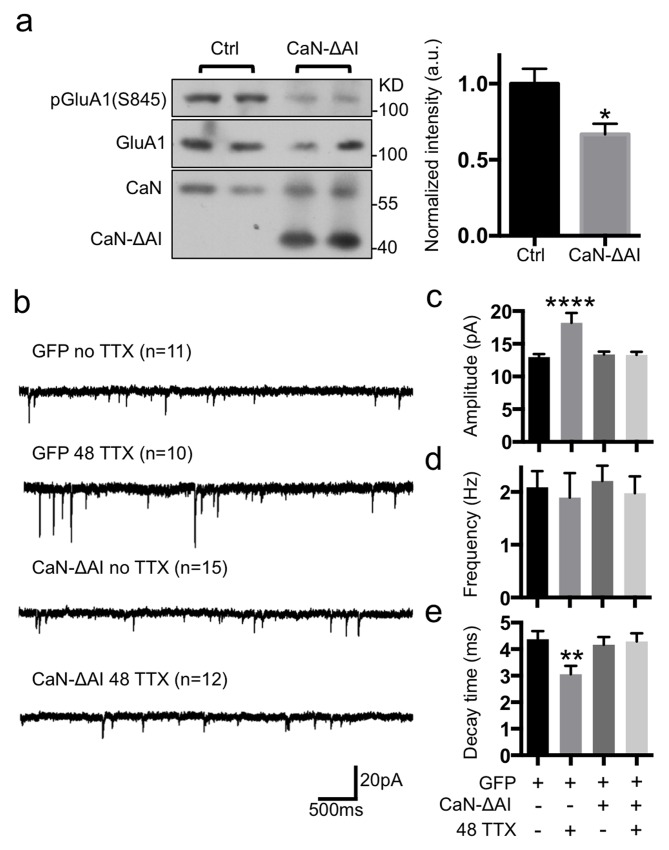
Constitutively active calcineurin mutant inhibited TTX-induced synaptic scaling. (a) Representative immunoblots showing overexpressing GluA1 with or without truncated mutant calcineurin (CaN-ΔAI) in HEK293 cells and a summary graph of pGluA1(S845) levels in each condition (*n* = 3 experiments, *p<.05, unpaired two-tailed Student's *t* test). (b) Representative traces of mEPSCs in each condition (*n* = number of cells). (c) Average mEPSC amplitude (****p<.0001, one-way ANOVA, uncorrected Fisher's LSD). (d) Average mEPSC frequency. (e) Average decay time (peak to 10%) (**p<.01, one-way ANOVA, uncorrected Fisher's LSD).

To test whether persistent calcineurin activity could block TTX-mediated synaptic scaling, we generated a constitutively active calcineurin mutant, which has Ca^2+^-independent, constitutive phosphatase activity, by deleting the calcineurin autoinhibitory domain (CaN-ΔAI) [38]. As expected, when we cotransfected HEK293 cells with GluA1 and CaN-ΔAI, pGluA1(S845) levels were significantly lower (*p* = .0198) than in cells transfected with GluA1 alone (Ctrl) (Figure 6a). Although CaN-ΔAI decreased pGluA1(S845), surface GluA1 levels remained unaffected in cultured neurons (Figure S1a). When CaN-ΔAI was cotransfected with GFP into neurons and we measured mEPSCs after 48 h TTX treatment, we found that TTX was unable to induce synaptic scaling in the presence of CaN-ΔAI (Figure 6b–e). However, TTX treatment of neurons expressing GFP alone induced a typical CPAR-mediated synaptic scaling (Figure 6b–e) as seen previously, with increased mEPSC amplitude (no TTX, 12.98±0.47 pA and 48 TTX, 18.21±1.52 pA, *p*<.0001) (Figure 6c) and decreased decay time (no TTX, 4.37±0.31 ms and 48 TTX, 3.05±0.31 pA, *p* = .0072) (Figure 6e), whereas frequency of mEPSCs was not altered (Figure 6d). This suggested that a gain-of-function calcineurin mutant could inhibit TTX-induced synaptic scaling.

### Synaptic Scaling-Mediated Partial Homeostasis of Ca^2+^ Signals

**Figure 7:**
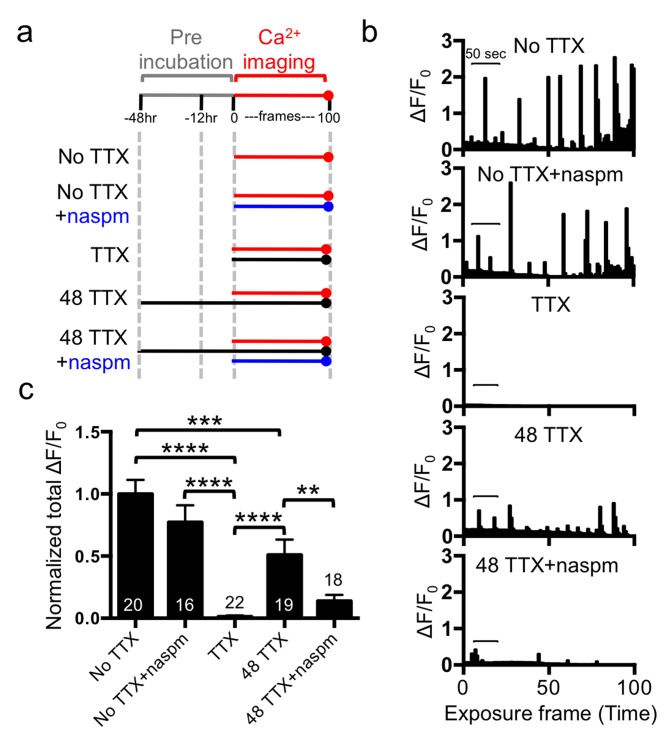
Synaptic scaling restored Ca^2+^ signals via TTX-induced CPARs. (a) Schematic of Ca^2+^ imaging protocol. A red line indicates Ca^2+^ imaging, a black line represents the presence of TTX, and a blue line shows the addition of naspm. (b) Example bar graphs of Ca^2+^ activity in each condition. Each bar represents the GCaMP5 fluorescence intensity detected in a single exposure frame. Scale bars are 50 s. (c) Normalized average of total Ca^2+^ activity in each condition (*n* = number of neurons, **p<.01, ***p<.001, and ****p<.0001, one-way ANOVA, uncorrected Fisher's LSD).

Ca^2+^ signals are thought to be important for synaptic scaling, which suggests that a reduction of Ca^2+^ influx may be a critical trigger for synaptic scaling [1],[19],[21],[22]. Furthermore, lowering cytoplasmic Ca^2+^ levels has been reported to enhance GluA1-containing AMPAR-mediated transmission [36]. We investigated Ca^2+^ activity in cultured neurons transfected with GCaMP5, a genetically encoded Ca^2+^ indicator [39] (Figure 7a). We found active spontaneous Ca^2+^ transients in neurons without TTX treatment (Figure 7b–c). To determine the effects of action potentials and mEPSC activity on Ca^2+^ transients, we first added TTX at the time of imaging and found that acute TTX treatment completely blocked Ca^2+^ activity (*p*<.0001) (Figure 7a–c). Furthermore, naspm treatment of neurons in the absence of TTX treatment had no significant effect on Ca^2+^ transients (Figure 7a–c), suggesting that action potentials play a critical role in generating the Ca^2+^ activity observed under these conditions, and that this activity is not dependent on CPARs. In contrast, following 48 h treatment with TTX, about 50% of the Ca^2+^ signal was restored (*p* = .0002), and this restored activity observed in the presence of TTX was significantly reduced by naspm (*p* = .0079) (Figure 7a–c). This suggested that TTX-induced scaling provided a mechanism for maintaining Ca^2+^ activity that was dependent in part upon the synaptic expression of CPARs.

**Figure 8:**
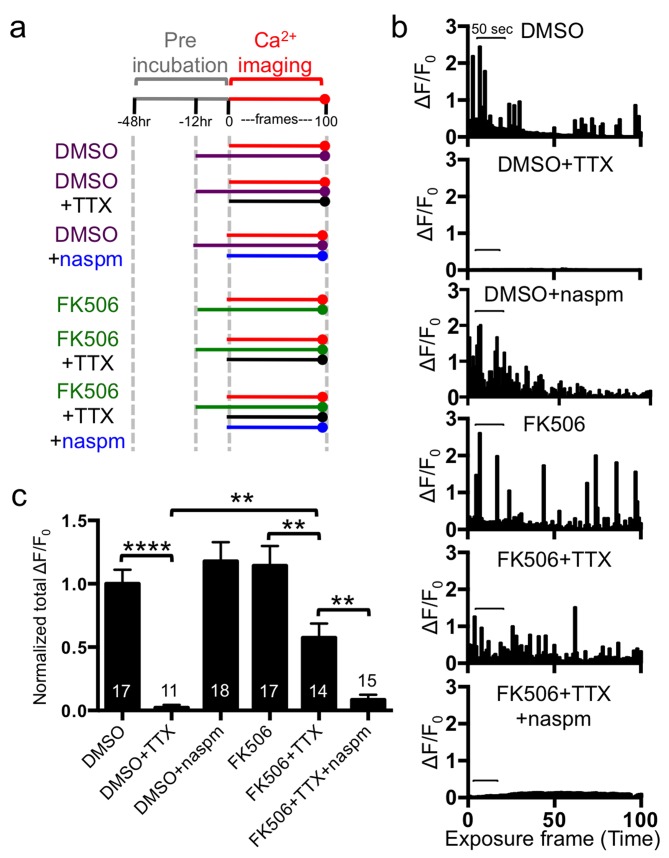
Synaptic scaling restored Ca^2+^ signals via FK506-induced CPARs. (a) Schematic of Ca^2+^ imaging protocol. A red line indicates Ca^2+^ imaging, a purple line shows the presence of DMSO, a black line represents the addition of TTX, a blue arrow shows the treatment of napsm, and a green line reveals the incubation of FK506. (b) Example bar graphs of Ca^2+^ activity in each condition. Each bar represents the GCaMP5 fluorescence intensity detected in a single exposure frame. Scale bars are 50 s. (c) Normalized average of total Ca^2+^ activity in each condition (*n* = number of neurons, **p<.01 and ****p<.0001, one-way ANOVA, uncorrected Fisher's LSD).

We next investigated effects of calcineurin inhibition on Ca^2+^ signals (Figure 8a). Neurons with 12 h DMSO treatment displayed normal Ca^2+^ activity, and acute TTX treatment completely inhibited the activity (*p*<.0001) (Figure 8a–c). Neurons treated for 12 h with FK506 showed active spontaneous Ca^2+^ transients comparable to those in neurons without TTX treatment (Figure 8a–c). Conversely, when TTX was acutely added at the time of imaging to neurons that had been treated for 12 h with FK506, TTX was unable to block the Ca^2+^ signals completely (*p* = .005) (Figure 8a–c). To determine whether this Ca^2+^ signal activity was mediated by CPARs, naspm was added to neurons at the time of recording (Figure 8a). Naspm significantly reduced the activity (*p* = .0069), indicating it was from CPARs (Figure 8a–c). Furthermore, we confirmed that CaN-ΔAI blocked synaptic scaling-mediated recovery of Ca^2+^ signals (*p*<.0001) (Figure S1b), consistent with the finding that CaN-ΔAI inhibited TTX-induced synaptic scaling (Figure 6c). This suggested that calcineurin activity is important for both synaptic scaling and Ca^2+^ homeostasis mediated by CPARs, which partially restored Ca^2+^ signaling. Taken together, these results demonstrate that CPAR/calcineurin-dependent synaptic scaling provides a mechanism for homeostasis of Ca^2+^ signals in part as a homeostatic response to activity deprivation-induced inhibition of Ca^2+^ activity.

### Synaptic Scaling Induced Ca^2+^ Release from the ER and Maintained CREB Activity

**Figure 9:**
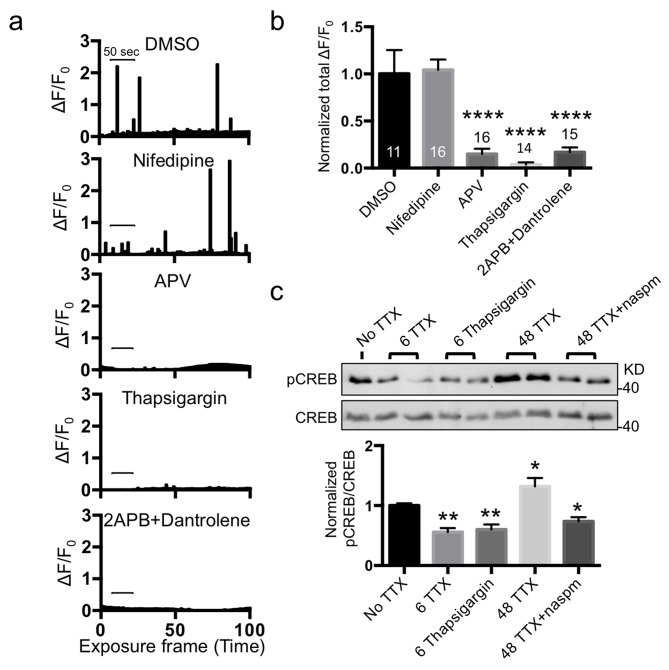
Synaptic scaling maintained CREB activity via ER Ca^2+^ release. (a) Example bar graphs of Ca^2+^ activity in each condition. Each bar represents the GCaMP5 fluorescence intensity detected in a single exposure frame. Scale bars are 50 s. (b) Normalized average of total Ca^2+^ activity in each condition (*n* = number of neurons, ****p<.0001, one-way ANOVA, uncorrected Fisher's LSD). (c) Representative immunoblots of nuclear fraction from each condition. A bar graph showing normalized ratio of pCREB/CREB in each condition (*n* = 3 experiments, *p<.05 and **p<.01, one-way ANOVA, uncorrected Fisher's LSD).

Both extracellular and intracellular sources of Ca^2+^ are used by neurons [40]. Although Ca^2+^ influx from extracellular sources is mediated by various Ca^2+^ channels including NMDA receptors (NMDARs) at synapses and voltage-gated Ca^2+^ channels in the plasma membrane, inositol 1,4,5-trisphosphate receptors (IP3Rs) and ryanodine receptors (RyRs) in the ER are responsible for intracellular Ca^2+^ release [40]. We first investigated which Ca^2+^ sources were responsible for GCaMP5-positive Ca^2+^ signals (Figure 9a). To address this question, we blocked each Ca^2+^ channel and measured spontaneous Ca^2+^ signals without drug pretreatment (Figure 9a–b). When we acutely treated neurons with 10 µM nifedipine, an L-type Ca^2+^ channel blocker, spontaneous Ca^2+^ signals were unaltered, but the NMDAR antagonist, 50 µM APV, significantly reduced Ca^2+^ activity (*p*<.0001), suggesting that GCaMP5 detected Ca^2+^ signals including those from NMDARs but not from L-type Ca^2+^ channels (Figure 9a–b). We next depleted Ca^2+^ from the ER by inhibiting sarco/endoplasmic reticulum Ca^2+^-ATPase using 1 µM thapsigargin and found that thapsigargin treatment completely inhibited Ca^2+^ activity (*p*<.0001) (Figure 9a–b). Moreover, blocking both IP3Rs and RyRs by 50 µM 2APB and 25 µM dantrolene significantly lowered Ca^2+^ signals (*p*<.0001), suggesting that GCaMP5 detected Ca^2+^ released from the ER, possibly dependent on the activity of NMDARs (Figure 9a–b). This further suggested that GCaMP5-positive Ca^2+^ signals restored by synaptic scaling were mediated by ER Ca^2+^ release.

NMDAR-mediated synaptic Ca^2+^ influx evokes Ca^2+^ signals in the nucleus via Ca^2+^ wave propagation through the ER [40]. This Ca^2+^ signaling is essential for synaptic plasticity and regulates gene expression through CREB in addition to local signaling in synapses [40]. Because an NMDAR antagonist blocks CREB activation [40] and CPARs also regulate ER Ca^2+^ release [41], we hypothesized that CPARs replace the role of NMDARs in synapse-to-nucleus Ca^2+^ signaling via the ER Ca^2+^ release when neuronal activity is chronically suppressed by TTX. Consistent with previous findings [42],[43], we found that CREB activity (assayed by measuring phosphorylation at serine 133 of CREB) was reduced with 6 h treatment of 2 µM TTX (*p* = .0004) or 1 µM thapsigargin (*p* = .0019), confirming that CREB activity was dependent on both neuronal activity and ER Ca^2+^ (Figure 9c). However, after synaptic scaling was induced by 48 h treatment with TTX, CREB activity was significantly increased, suggesting that ER Ca^2+^ signals restored by synaptic scaling provided a means to maintain CREB phosphorylation in the nucleus (Figure 9c). Treatment with 20 µM naspm for 6 h significantly reduced the CREB phosphorylation seen in neurons pretreated with TTX for 48 h (*p*<.0001), suggesting CPARs were responsible for homeostasis of CREB phosphorylation (Figure 9c). Taken together, this work shows that when neuronal activity is suppressed by TTX, synaptic scaling maintains basal CREB activity via synapse-to-nucleus Ca^2+^ signals by expression of CPARs at synapses and by ER Ca^2+^ waves.

## Discussion

**Figure 10:**
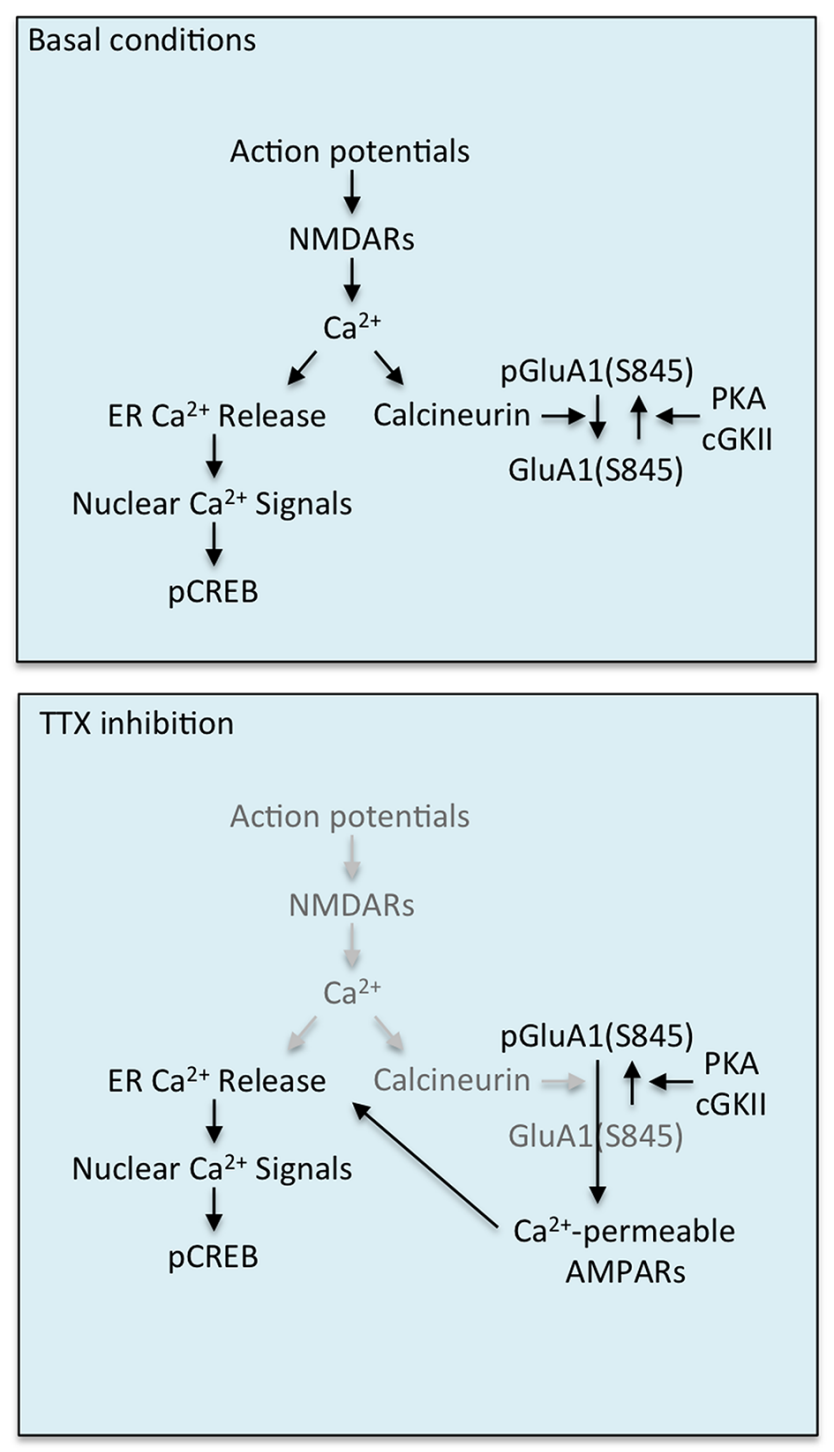
A model of CPAR/calcineurin-dependent synaptic scaling for homeostasis of Ca^2+^ signaling. A model showing regulation of synaptic insertion of CPAR by calcineurin leading to activity deprivation-induced synaptic scaling, followed by homeostasis of CREB activation in cultured cortical neurons.

We demonstrate a novel Ca^2+^ homeostasis-dependent mechanism of synaptic scaling mediated by calcineurin and CPARs. Based on our findings, we propose the following model. Under basal conditions, action potentials provide synaptic Ca^2+^ signals via NMDARs, followed by Ca^2+^-induced Ca^2+^ release from the ER, leading to nuclear Ca^2+^ signals that maintain CREB-mediated transcriptional activity. In addition, synaptic Ca^2+^ influx activates calcineurin, which removes GluA1 from the synaptic membrane by dephosphorylating pGluA1(S845), providing a balance between GluA1 insertion by kinases and removal by phosphatases in synapses (Figure 10). However, under the condition of activity deprivation, NMDAR-mediated synaptic Ca^2+^ influx is inhibited, leading to inactivation of calcineurin. This induces synaptic expression of CPARs via stabilization of pGluA1(S845), thereby enhancing synaptic strength and promoting synaptic Ca^2+^ influx via CPARs instead of NMDARs (Figure 10). This restores Ca^2+^ signals and CREB phosphorylation and activation. We thus suggest that synaptic scaling not only maintains neuronal activity by increasing CPAR-dependent postsynaptic strength but also maintains CREB activation by synapse-to-nucleus Ca^2+^ signaling.

Although it has been shown that postsynaptic AMPARs play a critical role in homeostatic synaptic plasticity, there is no generally agreed mechanism for synaptic scaling, possibly due to the fact that multiple experimental conditions have been investigated [13]. Many studies conducted in several experimental models support a role for this plasticity mediated by GluA1-containing AMPARs. For example, various protocols have been used to inhibit neuronal activity and induce synaptic scaling in cultured neurons, such as inhibition of action potentials by TTX [23], AMPARs by NBQX [21], L-type Ca^2+^ channels by nifedipine [21], or NMDARs and action potentials together by APV and TTX [22]. Regardless of inhibition protocols, each treatment induced CPAR-dependent synaptic scaling. Furthermore, visual deprivation in the cortex is sufficient for inducing CPAR-dependent homeostatic synaptic plasticity *in vivo* [26]. Nonetheless, the cellular mechanism by which neurons detect activity deprivation and what is the molecular readout of this signal that regulates postsynaptic AMPARs for synaptic scaling has not yet been identified. A recent study by Gainey et al. employing GluA2 knockdown reports that GluA2 is required for homeostatic synaptic plasticity [44]. It is possible that the increased expression of synaptic CPARs that occurs in the GluA2 knockdown prior to addition of TTX increases synaptic Ca^2+^ fluxes that prevent further CPAR synaptic trafficking required for synaptic scaling. In contrast, the GluA2 knockout exhibits normal synaptic scaling after chronic TTX treatment [45]. Significantly, in the knockout of GluA2, GluA1 levels at synapses are lower than in the wild type [46], a change which would generate significantly lower Ca^2+^ flux than the knockdown, which in turn would make synaptic scaling possible. Given these considerations, the experimental findings of the others are consistent with the current work.

Ca^2+^ influx in response to synaptic stimulation or action potentials plays an important role in regulating various neuronal functions including releasing neurotransmitter, modulating ion channels, and promoting synaptic plasticity and gene expression [47],[48]. Somatic Ca^2+^ levels are thought to be an important activity sensor in homeostatic synaptic plasticity [1],[19],[49]. Downstream effectors of Ca^2+^ signaling including CaMKs and adenylyl cyclases can be molecular readouts of the Ca^2+^ influx [13]. Because chronic neuronal inactivation reduces Ca^2+^ influx and downregulates adenylnly cyclases [50], cAMP-dependent PKA activity is unlikely elevated by TTX to increase phosphorylation of S845 of GluA1, although it needs further investigation. Calcineurin is the only Ca^2+^/calmodulin-activated phosphatase in the brain, and it is a major regulator of several key proteins mediating synaptic transmission and neuronal excitability in both pre- and postsynaptic areas [51]. Due to the fact that calcineurin inhibition promotes an increase in both mEPSC frequency and amplitude, it has been proposed to have a role in homeostatic synaptic plasticity [33]. Moreover, lowering basal Ca^2+^ levels has been shown to strengthen AMPAR-mediated transmission, which is dependent on GluA1 and calcineurin [36]. A computational modeling study predicts that calcineurin can be active at moderate Ca^2+^ concentrations, whereas the activity of PKA requires high Ca^2+^ levels [52]. It is thus possible that calcineurin can remain active in the short term, even after action potentials and synaptic Ca^2+^ influx are abolished by TTX. Consistent with this study, we found that calcineurin activity was not reduced immediately after TTX treatment, and the reduction was found after a 24 h treatment with TTX (Figure 3). This persistence of activity may explain why a significant length of time of application of TTX is required to express synaptic scaling. It has been shown that calcineurin inhibition increases pGluA1(S845) and selectively increases synaptic expression of CPARs [17]. Further investigation is required to determine how calcineurin inhibition selectively increases CPARs, given that it could potentially affect both GluA1 homomeric and GluA1/2 heteromeric AMPARs. We also showed that CaN-ΔAI was sufficient for reducing pGluA1(S845) levels, although surface GluA1 levels were not altered, which accounts for normal mEPSCs (Figure 6 and Figure S1a). Although it is not clear how normal levels of surface GluA1 are maintained when S845 phosphorylation is decreased, this is not surprising because several lines of studies already show that (1) there is normal synaptic transmission in the hippocampus of CaN-ΔAI overexpressed transgenic mice [53]; (2) in GluA1 S845A mutant mice, surface GluA1 levels are not affected [28]; and (3) GluA1 S845A mice also display normal mEPSCs in the amygdala [54]. Taken together, phosphorylation of GluA1 to yield pGluA1(S845) may not be critical for maintaining basal synaptic transmission, but can be important for activity-dependent plasticity, such as long-term potentiation or synaptic scaling.

It has been shown that lowering calcineurin activity with cyclosporin A, another calcineurin inhibitor, decreases not only enzymatic activity but also calcineurin protein levels [55], consistent with our findings (Figures 2a and 5a), suggesting that inactive calcineurin may be degraded. Neuronal activity regulates synaptic proteins and signaling through the ubiquitin-proteasome system, providing a mechanism that links activity and protein turnover [56]. Ca^2+^ entry is an important process regulated by neuronal activity that promotes a decrease of protein ubiquitination in synapses, which depends on calcineurin activity [57]. Calcineurin also can be ubiquitinated and undergo proteolysis in cardiomyocytes [58]. Thus, it is possible that chronic inhibition of neuronal activity decreases calcineurin activity by lowering Ca^2+^ influx, which promotes increased protein ubiquitination, including ubiquitination of calcineurin itself, followed by proteasome-mediated degradation, although this requires further investigation.

During activity deprivation, synaptic Ca^2+^ influx is reduced, possibly followed by inhibition of downstream Ca^2+^ signaling [1],[6],[12]. Synaptic scaling may provide a mechanism to overcome these problems. GluA1-containing CPARs are an attractive candidate for restoration of Ca^2+^ activity during synaptic scaling because unlike GluA2-containing, Ca^2+^-impermeable AMPARs, they not only have larger single channel conductance but also are Ca^2+^-permeable [59]. Based on our findings, we suggest that during homeostatic synaptic scaling, CPARs are stabilized in synapses and conduct Ca^2+^, which increases synaptic strength and also partially restores suppressed synaptic Ca^2+^ signals as shown by the finding that GCaMP5 predominantly detected Ca^2+^ release from the ER (Figures 7–9). In addition, cytosolic Ca^2+^ levels may reflect neuronal activity on a cell-wide basis, permitting Ca^2+^-dependent mechanisms to control all synapses, a feature of multiplicative homeostatic synaptic plasticity. Therefore, recruitment of CPARs may provide feedback regulation to maintain neuronal activity and Ca^2+^ signaling during synaptic scaling.

CaMKs are important for Ca^2+^-dependent synaptic plasticity, and reduction of CaMKIV activation is sufficient for inducting synaptic scaling without TTX treatment [19],[20]. CaMKIV-mediated activation of the CREB transcription factor is important for synaptic plasticity and learning [40]. Thus, it is possible that inhibition of CaMKIV activity reduces CREB activation and promotes synaptic scaling. Because the neuronal CREB transcription factor regulates various signaling pathways including those for learning, addiction, and pain [40], homeostasis of basal CREB activity can be important for brain function and may be maintained by synaptic scaling. Thus, synaptic scaling can provide a mechanism for maintaining basal levels of CREB-mediated transcriptional activity via synaptic Ca^2+^ and CaMKIV when neuronal activity is suppressed. Our data support this idea by showing that (1) TTX inhibited somatic Ca^2+^ signals, (2) TTX treatment reduced CREB phosphorylation, and (3) synaptic scaling restored Ca^2+^ signaling and CREB activation.

In summary, we conclude that synaptic scaling not only maintains neuronal stability by increasing CPAR-dependent postsynaptic strength but also maintains basal CREB transcriptional activity through nuclear Ca^2+^ signaling as a homeostatic response to suppression of neuronal activity.

## Materials and Methods

### Mouse Cortical Neuron Cultures and Neuronal Transfection

Cortical primary neurons were prepared by a modification of the previously described method [60]. Neurons were isolated from embryonic day 17–18 C57Bl6 or GluA1 S845A mouse embryonic brain tissues. All animal studies were performed with an approved protocol from New York University Langone Medical Center's Institutional Animal Care and Use Committee. Neurons were plated on poly-L-lysine–coated 15 cm dishes for biochemical experiments, size 12 mm cover glasses for electrophysiology and FRET assay, or glass-bottom dishes for Ca^2+^ imaging. Cells were grown in Neurobasal medium with B27 and 0.5 mM Glutamax (Life Technologies). For neuronal transfection, DIV4 neurons were transfected with Lipofectamine 2000 (Life Technologies) according to the manufacturer's protocol, and analysis was performed at DIV14–17.

### Generation of Constitutively Active Calcineurin Mutant

Constitutively active calcineurin mutant (CaN-ΔAI) was generated according to the previous study [38]. A stop codon was introduced at lysine 399 of wild-type murine calcineurin alpha (Addgene, 17871) to produce CaN-ΔAI that lacked the calmodulin-binding and autoinhibitory domains, leading to Ca^2+^-independent, constitutive phosphatase activity [38]. CaN-ΔAI was cotransfected with GluA1 into HEK293 cells using Lipofectamine 2000 (Life Technologies) to confirm expression and pGluA1(S845) levels by immunoblots. For surface GluA1 leveling, CaN-ΔAI was cotransfected with mCherry into neurons, and surface GluA1 was determined by incubation of a GluA1 antibody (Calbiochem, PC246) under the nonpermeable condition. The Alexa Fluor-488 secondary antibody (Molecular Probes, A-11008) was used to visualize surface GluA1, and proximal dendrites (<100 µm from the cell body) were captured using Zeiss Axiovert 200 m. Images were analyzed by the ImageJ software.

### Miniature EPSC Recording

Miniature EPSCs were measured in cortical neurons cultured from C57Bl6 or GluA1 S845A embryos at DIV14–17 as described previously [60]. Neurons were voltage clamped with the whole cell ruptured path technique during the recording. The bath solution contained (in mM) 119 NaCl, 5 KCl, 2.5 CaCl_2_, 1.5 MgCl_2_, 30 glucose 20 HEPES (Life Technologies), and 0.001 glycine (Sigma), pH 7.4. Patch electrodes (5–8 MΩ) were filled with (in mM) 120 K-gluconate (Sigma), 9 NaCl, 1 MgCl_2_, 10 HEPES, 0.2 EGTA (Sigma), 2 Mg-ATP (Sigma), and 0.2 GTP (Sigma). We added 1 µM TTX (Tocris Biosciences) and 10 µM bicuculline (Tocris Biosciences) to the bath to inhibit action potentials and miniature inhibitory postsynaptic currents, respectively. mEPSCs were recorded at −60 mV with a Warner amplifier (PC-501A) and filtered at 1 kHz. Recordings were digitized (Digidata 1440, Molecular Devices) and analyzed using the Mini Analysis software (Synaptosoft). The access resistance (R_a_<25 MΩ) was monitored during recording to eliminate artifacts. Events whose amplitude was less than 7.5 pA were rejected. To induce synaptic scaling, neurons were pretreated with 2 µM TTX for 48 h or 5 µM FK506 or DMSO for 12 hrs. We added 20 µM naspm (1-naphthylacetyl spermine trihydrochloride, Tocris Biosciences) or 5 µM philanthotoxin-74 (Tocris Biosciences) to suppress CPAR-mediated transmission in the bath solution. For the CaN-ΔAI experiment, GFP was cotransfected with CaN-ΔAI to visualize transfected neurons, and mEPSCs were analyzed at DIV14–17.

### Synaptosome Purification, Surface Biotinylation, and Immunoblots

Synaptosomal fractions from DIV14 primary cortical neurons were prepared as described previously [60],[61]. Surface biotinylation was performed according to the previous study [60]. Equal amounts of protein were loaded on 10% SDS-PAGE gel and transferred to the nitrocellulose membrane. Membranes were blotted with GluA1 (Millipore, 1∶5,000), GluA2/3 (Millipore, 1∶500), pGluA1(S845) (Millipore, 1∶1,000), calcineurin (Millipore, 1∶1,000 or Santa Cruz Biotechnology, 1∶500), actin (Sigma, 1∶5,000), pCREB (Cell Signaling, 1∶500), and CREB (Cell Signaling, 1∶1,000) antibodies and developed with ECL (Perkin Elmer). Synaptosomes were isolated from at least three independent cultures, and immunoblots were least duplicated for quantitative analysis.

### FRET-Based Calcineurin Activity Assay

Neurons were transfected with the calcineurin activity biosensor, and FRET activity was measured at DIV14 according to a modification of the previously described method [31]. Neurons were pretreated with 2 µM TTX for 12, 24, or 48 h, or 5 µM FK506 for 12 h, and fixed with 4% paraformaldehyde. Images were captured by using Applied Precision PersonalDV live-cell imaging system in the Microscopy Core of New York University Langone Medical Center. The following formula was used to calculate the emission ratio:
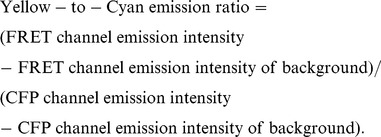
Pseudocolor images of the emission ratio were generated by using the ImageJ software, as previously reported [62].

### Ca^2+^ Imaging

DIV4 neurons were transfected with GCaMP5 (Addgene, 31788). Neurons were grown for 10–12 d after transfection in Neurobasal medium without phenol red and supplemented with B27 and 0.5 mM Glutamax. Glass-bottom dishes were mounted on a temperature-controlled stage on Zeiss Axiovert 200M and maintained at 37°C and 5% CO_2_ using a Zeiss stage incubator model S with CTI, digital temperature, and humidity controller. The imaging was captured for periods of 0.5 to 1.0 s depending on the intensity of the fluorescence signal using a 63× oil-immersion objective. One hundred images were obtained with a 1-s interval, and Ca^2+^ activity in the cell body (excluding dendrites) was analyzed using the ImageJ software. F_0_ was determined as the minimum value during the imaging. Total Ca^2+^ activity was obtained by combining 100 values of ΔF/F_0_ = (F_t_−F_0_)/F_0_ in each image, and values of ΔF/F_0_<0.3 were rejected due to bleaching.

### Nuclear Extract Preparation

Neurons pretreated with 2 µM TTX for 6 or 48 h or 1 µM thapsigargin for 6 h were lysed with a nuclear preparation buffer A (10 mM Tris-HCl, pH 7.9, 1.5 mM MgCl_2_, 10 mM KCl, and 0.25% NP40). Nuclear fraction was collected by centrifugation, resuspended in a nuclear preparation buffer B (20 mM Tris-HCl, pH 7.9, 1.5 mM MgCl_2_, 420 mM KCl, 0.2 mM EDTA, and 20% glycerol), and analyzed by immunoblots.

### Statistics

Most statistical comparisons were analyzed with the GraphPad Prism6 software. Unpaired two-tailed Student's *t* tests were used in single comparisons. For multiple comparisons, we used one-way analysis of variance (ANOVA) followed by Fisher's Least Significant Difference (LSD) test to determine statistical significance. The Kolmogorov-Smirnov (K-S) test (http://www.physics.csbsju.edu/stats/KS-test.html) was used for comparisons of cumulative probabilities. Results were represented as mean ± s.e.m., and a *p* value<.05 was considered statistically significant.

## Supporting Information

Figure S1 Effects of CaN-ΔAI on surface GluA1 and Ca^2+^ activity. (a) Representative images of GluA1 surface labeling (green) and mCherry were used as markers for transfection (red) (*n* = number of cells). Scale bar is 2 µm. A summary graph of surface GluA1 levels in each condition. (b) Example bar graphs of Ca^2+^ activity in each condition. Each bar represents the GCaMP5 fluorescence intensity detected in a single exposure frame. Scale bars are 50 s. Normalized average of total Ca^2+^ activity in each condition (*n* = number of neurons, **p<.01 and ****p<.0001, one-way ANOVA, uncorrected Fisher's LSD).
(TIF)

## Abbreviations

2APB: 2-Aminoethoxydiphenyl borate
AKAP: A-kinase anchoring protein
AMPA: α-Amino-3-hydroxy-5-methyl-4-isoxazolepropionic acid
APV: (2*R*)-amino-5-phosphonopentanoate
CaMK: Ca^2+^/calmodulin-dependent protein kinase
cAMP: cyclic adenosine monophosphate
CFP: cyan fluorescent protein
cGKII: cGMP-dependent protein kinase II
cGMP: cyclic guanosine monophosphate
CPAR: Ca^2+^-permeable AMPARs
CREB: cAMP response element-binding protein
DIVs: days in vitro
ER: endoplasmic reticulum
FRET: fluorescence resonance energy transfer
IP3R: inositol 1,4,5-trisphosphate receptor
mEPSC: miniature excitatory postsynaptic current
NASPM: 1-naphthyl acetyl spermine
NFAT: nuclear factor of activated T cells
NMDA: *N*-Methyl-D-aspartic acid
PhTX: philanthotoxin-74
PKA: cAMP-dependent protein kinase A
RyR: ryanodine receptors
SAP97: synapse-associated protein 97
TTX: tetrodotoxin
YFP: yellow fluorescent protein
