# Supplementary figures and images for: Calcineurin Mediates Synaptic Scaling Via Synaptic Trafficking of Ca2+-Permeable AMPA Receptors
Source: PLoS Biol. 2014 Jul 1;12(7):e1001900. doi: 10.1371/journal.pbio.1001900 (PMC4077568; doi:10.1371/journal.pbio.1001900)

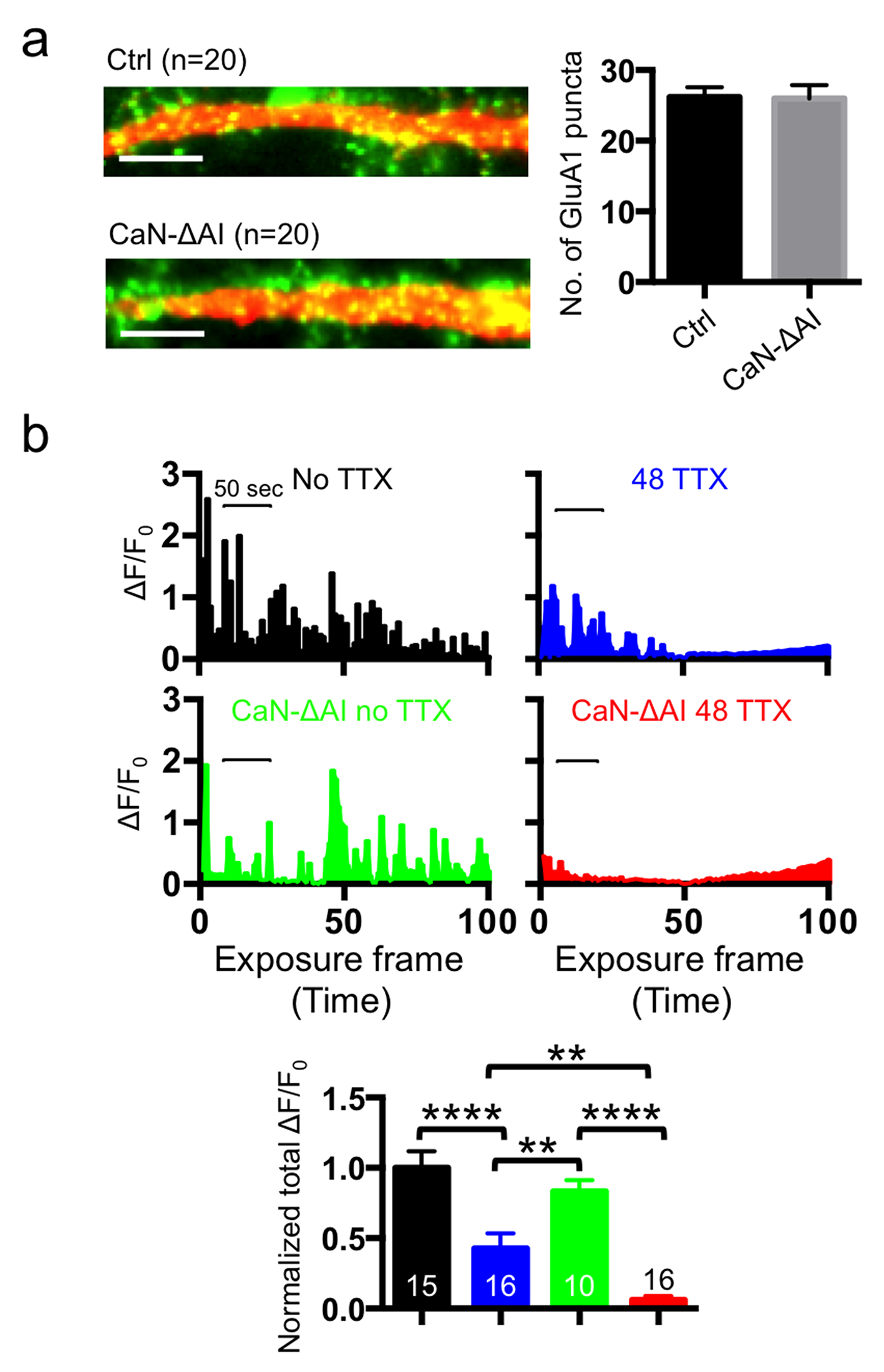

Supplement: Figure S1 — Effects of CaN-ΔAI on surface GluA1 and Ca2+ activity. (a) Representative images of GluA1 surface labeling (green) and mCherry were used as markers for transfection (red) (n = number of cells). Scale bar is 2 µm. A summary graph of surface GluA1 levels in each condition. (b) Example bar graphs of Ca2+ activity in each condition. Each bar represents the GCaMP5 fluorescence intensity detected in a single exposure frame. Scale bars are 50 s. Normalized average of total Ca2+ activity in each condition (n = number of neurons, **p<.01 and ****p<.0001, one-way ANOVA, uncorrected Fisher's LSD). (TIF) [file pbio.1001900.s001.tif]
